# Indications for Extraction of the Teeth

**Published:** 1871-08

**Authors:** J. F. Johnston

**Affiliations:** Indianapolis


### THE DENTAL REGISTER.

Vol. XXV.] AUGUST, 1871. [No. 8.

### Communications.

### INDICATIONS FOR EXTRACTION OF THE TEETH.

BY J. F. JOHNSTON, D. D. S., INDIANAPOLIS.

**Read before the Indiana State Dental Association, at Fort Wayne, June 27, 1871.**

The Executive Committee have proposed this question: "What are the conditions of a tooth which will justify its extraction?"

In this paper we purpose to discuss this subject as though the question had been worded thus: What are the conditions which will justify the extraction of a tooth?

We are all aware that certain conditions other than the immediate disease of a tooth, often renders extraction not only justifiable, but absolutely necessary. Not being able to see how an intelligent system of treatment of a diseased tooth, frequently complicated with an inflamed condition of the surrounding tissues, and not infrequently in the case of females, aching in sympathy with some abnormal condition of the uterine organs, or in cases of pregnancy, can be pursued, without carefully studying the condition of the patient—taking *at least,* all the parts *involved* under consideration. We shall take the privilege of giving ourself such latitude in its investigation, as in our judgment a profitable examination of the question may require. To simply discuss how much of the substance of a tooth may be wasted and destroyed by decay, and yet be restored to near its original proportions under the skillful manipulations of the dentist, or per contra, how much of it must be gone before the remains may be justifiably taken away, would not in our opinion come up to the expectations nor meet the desire of the authors of this question. To follow it up then, and examine its other phases of disease, besides simply caries, we must go further than the original question would indicate.

As is well known, the older authors and practitioners of their day advocated the extraction of teeth for what would now be considered very trifling and entirely insufficient causes. On looking back at the condition of our profession in their day, we can but rejoice at the progress we have made in the cure, as well as prevention of disease. The medical profession, ennobled for its good works, and usually ever on the alert to save from disease, have been singularly careless in their observation of the teeth. Even in our day, they have been in the habit of extracting teeth without questioning, for almost any trifling ailment, using many times but little care, frequently removing the wrong tooth,—all this owing to the fact, that the proper *value* had not been placed upon them; but now, they too, we are pleased to note, with comparatively few exceptions, are alive to the importance of preserving the teeth, and advise accordingly.

Leonard Kœcker, in his work, the "Principles of Dental Surgery," (from the perusal of which by the way, the reader will infer the author lived on very good terms with himself, but who has, nevertheless, said many good things,) represented perhaps very nearly the status of dentistry both in this country and in England, about fifty years ago. Whilst extraction is spoken of by him as *the* treatment ordinarily for teeth in which the pulp is exposed, and in cases of simple alveolar abscess, it is yet quite gratifying to learn that he successfully treated cases where the nerve *was* exposed, and also other morbid conditions. But his modesty did not prevent him from representing himself as an advanced practitioner of that time, and no doubt he was. It will perhaps not be uninteresting to extract an item from case No. XVI, in the treatment of a young lady's teeth in the year 1818, which will tend to give us an idea of the state of our profession about that time—there is nothing extraordinary in the extract, it is merely the *fact* we wish to note—he says: "The caries was extirpated in two places, and the cavities were filled with gold, after the lining membrane of one which had become exposed, had been properly treated." Indeed, the successful treatment of the exposed nerve is frequently spoken of by him, but we regret to say the mode of treatment is not given, we are left in the dark as to whether the nerve was destroyed or saved. His work being intended as much for the general as professional reader, is perhaps the cause of this omission, though the dental profession of that day do not appear to have been *anxious* to communicate or make public their treatment of cases. It is gratifying, however, to know that they were extending their researches and making successful efforts to save a class of teeth, that had been so generally sacrificed. It also leads us to think that our progress in this direction, though very commendable, has not been so marvelously rapid, as we are often led to believe.

The late Chapin A. Harris, assigns among many other indications for the extraction of teeth, familiar to all of you, and the most of which we can indorse, the following from which we as a rule must dissent: "A constant discharge of feted matter through a carious opening in the crown from the nerve cavity, and from the canal of the root of a tooth." Also, "a tooth which is the cause of abscess in its alveolus, should not, as a *general rule*, be permitted to remain in the mouth." We, in this day, as a *rule*, believe in the successful treatment and preservation of such teeth. Professor Taft, in his excellent work on "Operative Dentistry," says: "Alveolar abscess, terminating on the outside of the face, or tending to it, *always* indicates the removal of the offending tooth," also, "ulceration of the investing membrane clearly points to extraction as the remedy." We agree with him fully and unreservedly in the first and last instances, and in the second also, unless it be found that by timely lancing inside the mouth, and the introduction of a rope of cotton or other suitable material into the opening thus made, the abscess may be drained of its contents and the parts restored to health. The tooth being treated for filling, as in other cases of alveolar abscess. Failing to accomplish this, of course, the tooth should be extracted in time to prevent its causing a disgusting ulcer upon the outer surface of the cheek; leaving an unsightly scar after the operation of removal has taken place, which must inevitably precede a cure sooner or later.

Tomes, in his admirable work, would seem to regard extraction as justifiable in several cases in which many of our more ardent and enthusiastic practitioners would not perhaps acquiesce, as for instance, in acute inflammation of the dental pulp, while stating that it is susceptible of successful treatment. Yet he says, "but should it be found that the pulp of the tooth is passing into a state of disorganization," etc., etc., the removal of the tooth offers the only certain and speedy means of terminating the disease, and in polypus of the pulp "preservative treatment, says he, will fail, owing to a coincident enlargement of the aperture at the extremity of the root, and to a morbid condition of the vessels and other tissues to which that aperture gives passage, and again in thickening of the periosteum, "this bulbous state of the dental membrane," (often termed fungous,) is occasionally found in connection with dental exostosis, and sometimes with necrosis, but in the latter case, the disease is disposed to become active, and to end in alveolar abscess, there is but one method of treatment—the affected tooth should be removed." In the last named conditions our judgment and experience leads us to fully concur with the distinguished author. On our part, it is perhaps hardly necessary to add that extraction to correct certain cases of irregularity, to treat other diseased parts, as the antrum, necrosis of the jaw, or alveolus, etc., etc., would be justifiable, as this is the acknowledged remedy in such cases, being fully indorsed by all authors upon this subject. But there is a class of teeth we would especially direct attention to. We allude to the first, commonly called the sixth year molars. These teeth, as Professor Taft has shown in his tabular statement of the relative decay of the teeth—as exhibited in one thousand cases—see page 63, Operative Dentistry, are far more liable to decay than any others, the per centage being 37 to only 10½ in the third molars. In speaking of them, he says, "the first molars are much more liable to decay than any other teeth, since they are less perfectly developed than those formed at a later period of life." We all know from actual experience and observation, that this is literally true. This fact being established, would it not be sound sense and consequently good practice, to direct our efforts to the more perfect development of the third molars? We think it would; and with this end in view, we think that when a case is presented for treatment, if the first molars are found in a much crowded jaw, imperfectly formed, soft and decayed, upon each proximal surface, or softening and decaying in grooves near the margin of the gums, either on the buccal surface, which is most frequent, or on the lingual surface as sometimes happens; when this condition exists nearly as described, or they show other clear indications of rapid decay, in which all the first molars are more or less involved, that it would be far better for the future comfort and health of the patient, if they were removed—relieving thereby the lateral pressure upon the other over crowded teeth, which can afterwards be more perfectly cleansed, and providing room for the future development of the *dentes sapientiæ*, which we may reasonably hope and expect, owing to the patient's better care of the teeth usual at that age, and consequent improved physical condition, will come in, well formed, in good position, and with reasonable care, perform an useful part in the mastication of food for many years, and perhaps the life of the patient—thus, far more than compensating for the loss of the already carious first molars that have been extracted. Under such circumstances, the loss of these teeth would not be observed, nor the symmetry of the mouth impaired, but on the contrary, in many instances improved. In such emergencies, nature would seem to be putting forth her best efforts in the development of the *dentes sapientiæ*, to meet, we could fancy, an anticipated deficiency in the jaws at that time, for without the removal of the first molars, there is seldom sufficient room for the perfect development of the third.

But, gentlemen, so much has been said of late years denunciatory and condemnatory of those who extract teeth for almost any cause, and so many practitioners have expressed themselves as having suffered so much mental anguish from their torturing consciences, visions of slaughtered teeth, like Banquo's ghost ever rising up before them, that it requires a little courage to advocate extraction under almost any circumstances. So inclined we are, it appears to us, to run into extremes, that the result has been that thousands of old, offensive, irritating, worthless snags, have been patched up, with all sorts of amalgams and other vile compounds, resulting in injury to the other teeth and gums, if not to the general health, and which of course, ought to have been promptly extracted. This is the misfortune of following too much the single idea, as the dentist? (if we may call him one) does, who extracts everything to make way for his beautiful artificial teeth; and not going thoughtfully far enough into the investigation of each case, or else following blindly as we fear many do, the teachings of some dental luminary, their beau ideal. We have observed of late years, many mouths in which the condition just referred to existed, that would have been in a much more healthy, comfortable, and useful state, if some of the teeth had been removed. In such cases especially, a sound, enlightened judgment is required. Here it is that something in addition to and more than mere manipulative skill is needed. Of this class of cases, the attempted preservation of the first molars under disadvantageous circumstances, are most frequently met with. We conceive it to be quite impossible, however, to describe so accurately any condition of a tooth, that may serve as an undeviating rule in all cases. Often a tooth that appears well worth an effort to save, owing to the condition of its neighbors, the surrounding soft tissues, the age or peculiar idiosyncracy of the patient, it will be found best to remove. As stated at the outset, we have not attempted in this paper to describe just those conditions that would render extraction absolutely necessary, as viewed by the light we possess, for this, as before intimated, must ever remain a matter of intelligent judgment with each individual operator. Nor have we attempted to describe how much diseased a tooth may be and yet be saved, because this also depends not only upon so many contingencies, but very much upon the manipulative and medical skill in the treatment, as well as the capacity to determine its *real* condition. Cases appearing to one hopeless, which to another of better parts are comparatively easily managed. We have preferred to speak of those general conditions of a class of teeth that, as a rule, to which there are exceptions—we thought it would be better to extract than attempt to save. We trust we shall not be understood as saying one word in disparagement of conservative dentistry; for we feel satisfied that no one can entertain a more profound contempt and utter loathing of that class, who sacrifice needlessly and remorselessly the natural teeth—either from motives of cupidity, ignorance, or careless indifference. Yet, we are free to say, that the great desire to save all teeth, almost without regard to their condition and the other parts immediately or remotely involved, though a noble aspiration on the part of the dentist, is yet a piece of enthusiasm that is in many instances carried farther than a more deliberate judgment, and future results would warrant. But at the risk of repetition, we will add, that the utmost we can do is to use our best judgment conscientiously, and in forming our opinion of a case, as for or against extraction, the age, the habits and moral and mental condition of the patient, should receive our consideration. The removal of a tooth should always be regarded as too serious a matter to be done hastily, unthinkingly, and without careful investigation. The patient's condition may be such as to render the operation one of great danger, and in such a condition, extraction should not be attempted, although appearing otherwise necessary, unless the danger to the patient would seem to be less in removing, than allowing the offending tooth for the present to remain.

In conclusion, having shown that the question is one that can not be definitely answered when so much depends upon the varying ability of the dentist, and the ever varying conditions of our patients, constitutionally, educationally, and it would perhaps not be out of place to add pecuniarily—for disguise it as you may, money has much to do in the successful treatment of disease—our best dentists not usually being the lowest in their fees, it will be seen that it procures the best talent, consequently the best remedies; provides the means of absolute rest and careful nursing, which is often indispensable to the treatment of a diseased tooth. These things can not be indulged in by the poor laboring man or woman. We are sorry for them—their lot is hard, but it is so. Even if the dentist was willing to give his services in such cases, (which frequently would be a greater sacrifice than he could afford to make,) such patients, could not afford to lose their time. Relief quick and decided, is what they want even at the sacrifice of the tooth, and it does not hurt our conscience to afford it in that way to this class, taking their habits and necessities into consideration, it is in our judgment the best service we can render them, and consequently, justifiable practice.

We can not conclude this paper in better words than those uttered by that eminent old pioneer teacher, the late Chapin A. Harris:

"Whenever a tooth can be restored to health, it should always be done, but tampering with such as can not be rendered healthy and useful, and which by remaining in the mouth, exert a deleterious influence, not only upon the adjacent parts, but also upon the general health, can not be too strongly deprecated."

